# Genetic Basis of Non-Syndromic Childhood Glaucoma Associated with Anterior Segment Dysgenesis: A Narrative Review

**DOI:** 10.3390/ph18091352

**Published:** 2025-09-09

**Authors:** Nicola Cronbach, Cécile Méjécase, Mariya Moosajee

**Affiliations:** 1 UCL Institute of Ophthalmology, London EC1V 9EL, UK; nicola.cronbach.23@ucl.ac.uk (N.C.); c.mejecase@ucl.ac.uk (C.M.); 2 Ocular Genomics and Therapeutics, The Francis Crick Institute, London NW1 1AT, UK; 3 Moorfields Eye Hospital NHS Foundation Trust, London EC1V 9EL, UK

**Keywords:** childhood glaucoma, anterior segment dysgenesis, gene editing, nonsense suppression, aniridia

## Abstract

Twenty causative genes have been reported that cause non-syndromic childhood glaucoma associated with anterior segment dysgenesis. *FOXC1*, *PAX6* and *PITX2* are the most well-known, but cases linked to *SLC4A11*, *PITX3* and *SOX11* have also been reported. As genetic testing becomes increasingly widespread and rates of molecular diagnosis rise, the extent of phenotypic overlap between the different genetic causes of non-syndromic glaucoma associated with anterior segment dysgenesis is becoming more evident. Taking aniridia as an example, whilst *PAX6* mutations remain the predominant cause, variants in *CYP1B1*, *FOXC1*, *PXDN* and *SOX11* have also been reported in patients with childhood glaucoma and aniridia. Developments in molecular-based therapies for retinal and corneal disease are advancing rapidly, and pre-clinical studies of gene-based treatments for glaucoma and aniridia are showing promising results. Use of adeno-associated viral vectors for gene delivery is most common, with improvements in intraocular pressure and retinal ganglion cell survival in Tg-*MYOC^Y437H^* mouse models of glaucoma, and successful correction of a germline *PAX6^G194X^* nonsense variant in mice using CRISPR-Cas9 gene editing. This review will explore the actions and interactions of the genetic causes of non-syndromic glaucoma associated with anterior segment dysgenesis and discuss the current developments in molecular therapies for these patients.

## 1. Introduction

**Figure 1:**
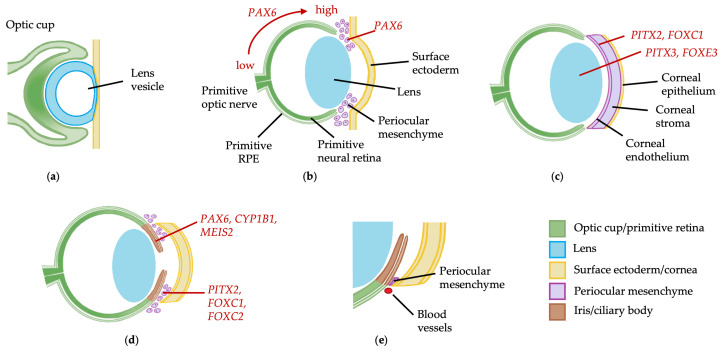
Anterior segment embryological development: (**a**) at day 37 of gestation, a double layered optic cup and lens vesicle can be seen; (**b**) at 47 days, the optic cup has formed primitive neural retina and retinal pigment epithelium with the optic stalk forming primitive optic nerve. Optic cup patterning is dependent on the *PAX6* expression gradient, from high expression at the peripheral optic cup tips down to low expression centrally. Cells of the central lens placode migrate to the posterior lens vesicle and elongate to form primary lens fiber cells, and the first wave of neural crest cells from the periocular mesenchyme migrates between the lens and surface ectoderm; (**c**) at 57 days, the primitive corneal epithelium is formed from the surface ectoderm, the corneal stroma and endothelium are formed from neural crest cells of the periocular mesenchyme, and there is separation of the lens stalk and formation of the anterior chamber; (**d**) by 15 weeks, elongation of optic cup tips along the anterior lens surface to form the iris and ciliary body epithelium occurs, and there is a second wave of periocular mesenchyme migration into the anterior chamber to form the iris and ciliary body stroma; (**e**) at 20 weeks, there is an accumulation of periocular mesenchyme in the iridocorneal angle which subsequently elongates and forms lamellae to become the trabecular meshwork, and vessels form in the adjacent sclera which will become Schlemm’s canal. Gene expression is marked in red. RPE, retinal pigment epithelium.

Ocular development commences at gestational day 21 with induction of the eye field in the anterior neural plate prior to lateral evagination of the optic vesicles through the cephalic mesenchyme [1,2]. At gestational day 27, the optic vesicle contacts the surface ectoderm, and signaling through bone morphogenetic protein (BMP) and retinoic acid first from the optic vesicle and then the lens placode stimulates invagination of both structures to form the optic cup and lens vesicle, which are present by day 37 (Figure 1a) [1]. By day 47, differentiation of the optic cup into primitive neural retina and retinal pigment epithelium (RPE) has commenced, and neural crest cells from the periocular mesenchyme begin to migrate into the developing anterior segment under the control of *PAX6* (Figure 1b) [3]. *PITX2* and *FOXC1*, expressed by the presumptive cornea, and *PITX3* and *FOXE3*, expressed by the developing lens, have roles in regulating differentiation of the periocular mesenchyme into corneal keratocytes and endothelium, and detachment of the lens vesicle from the cornea, respectively (Figure 1c) [2]. From gestational week 15, *PAX6*, *BMP4* and *OTX1* stimulate elongation of the peripheral tips of the optic cup, which express *CYP1B1* and *MEIS2*, along the anterior lens surface to form the epithelium of the ciliary body and iris (Figure 1d) [2,4,5,6]. Concurrently, a second wave of migration of the periocular mesenchyme into the anterior segment occurs, expressing *PITX2*, *FOXC1* and *FOXC2*, which will form the ciliary body and iris stroma [2,3,5]. Trabecular meshwork development occurs from gestational week 20 with elongation, flattening and separation of mesenchymal cells in the iridocorneal angle to form lamellae, accompanied by vascular development in the adjacent sclera which becomes Schlemm’s canal (Figure 1e) [2,7].

**Figure 2:**
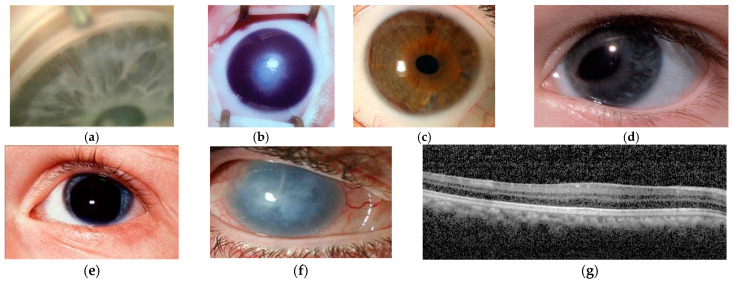
Clinical features of childhood glaucoma and anterior segment dysgenesis: (**a**) immature scalloped appearance of iridocorneal angle in primary congenital glaucoma; (**b**) central corneal opacity in Peters anomaly; (**c**,**d**) Axenfeld–Rieger anomaly: (**c**) iris hypoplasia and posterior embryotoxon; (**d**) corectopia; (**e**–**g**) congenital aniridia: (**e**) iris hypoplasia; (**f**) aniridia-associated keratopathy; (**g**) foveal hypoplasia.

Glaucoma is an overarching term for optic neuropathies resulting from retinal ganglion cell loss, which are typified by optic disc cupping and associated visual field defects. Childhood glaucoma, affecting individuals under 18 years of age, accounts for 5% of pediatric blindness worldwide [8]. In addition to optic nerve changes and visual field defects, features of childhood glaucoma include corneal enlargement and Haab striae, increased axial length and intraocular pressure >21 mmHg; two or more features are required for diagnosis [9]. Childhood glaucoma is classified according to the underlying cause and whether this is primary or secondary to another ocular or systemic condition [9]. Primary congenital glaucoma develops before 4 years of age and may be further divided into neonatal (0–1 month), infantile (>1–24 months) and late (>24 months) onset disease. It is a primary goniodysgenesis and presents with an immature appearance of the iridocorneal angle (Figure 2a) [9,10]. Juvenile open angle glaucoma presents after 4 years of age and with normal angle appearances. Secondary causes of childhood glaucoma include onset following cataract surgery, glaucoma associated with non-acquired ocular or systemic anomalies, and glaucoma associated with acquired conditions such as trauma and uveitis.

Anterior segment dysgenesis (ASD) is a broad term that incorporates developmental anomalies involving structures in the anterior segment of the eye [11,12,13]. These may affect a single structure, such as microcornea, or involve multiple structures, such as the iridocorneal and keratolenticular adhesions seen in Peters anomaly (Figure 2b). Between 30 and 75% of patients with ASD develop glaucoma depending on the subtype of dysgenesis [14,15], and young patients with either glaucoma or ASD should be thoroughly examined for features of each condition [13,15,16,17]. Many of the anterior segment dysgeneses have variable phenotypes with considerable overlap; for example, Axenfeld–Rieger anomaly (Figure 2c,d) may include corectopia, polycoria and iris hypoplasia in association with posterior embryotoxon, and like Peters anomaly, may also feature iridocorneal adhesions [15]. Patients with congenital aniridia (Figure 2e–g) present with underdevelopment of the iris of variable severity and foveal hypoplasia; some will also have lenticular opacities and optic nerve hypoplasia from birth, and many will develop glaucoma and aniridia-associated keratopathy by adulthood [14,18].

The molecular pathways involved in anterior segment development are highly complex. There is an overlap between the known genes that cause childhood glaucoma and those that cause anterior segment dysgenesis [11,12,13], and there is increasing evidence of digenic and likely polygenic influences on the development process, resulting in phenotypic variation. For example, mice with either *FoxC1*^+/−^ or *Cyp1b1*^−/−^ mutations who also carry the tyrosinase-deficient *Tyr^c–2J^* allele develop more severe anterior segment anomalies and angle defects, respectively [19]. In humans, *CYP1B1* mutations have been shown to be modified by *PITX2*, with *CYP1B1*-related juvenile glaucoma patients carrying the *PITX2^P179T^* allele developing earlier onset glaucoma than their *PITX2^wt^* relatives [20]. Similarly, glaucoma onsets at an earlier age in patients with *TEK* mutations modified by *SVEP1^R997C^* [21] and those with *MYOC* mutations modified by *COL15A1^R163H^* [22].

However, many of the factors that contribute to anterior segment development remain unknown. Molecular diagnostic rates for childhood glaucoma and anterior segment dysgenesis remain below 25% [12,23], indicating the need for further studies to investigate additional genes and biological pathways that influence this process. Whilst certain genes, such as *CYP1B1*, *PAX6* and *FOXC1*, have been identified in multiple patients across populations, others have been reported only in single cases or small case series. Robust cellular and animal models of disease aid in establishing the phenotypic features associated with different genetic variants and allow detailed morphological and functional studies to be undertaken. For example, the development of an *Adamts18*-knockout mouse model in 2016, coupled with developments in transcriptomic analysis, has enabled extensive investigation of its role in ocular and systemic development [24]. However, similar models have not yet been developed for all genes in which pathogenic variants causing non-syndromic childhood glaucoma associated with anterior segment dysgenesis have been identified.

The aim of this review is to summarize the functions, biological relationships and associated phenotypes of the known genes that have been identified in patients with non-syndromic childhood glaucoma associated with anterior segment dysgenesis and discuss potential avenues for future treatments, to provide a platform from which to expand our knowledge of this under-investigated area.

## 2. Results

**Figure 3:**
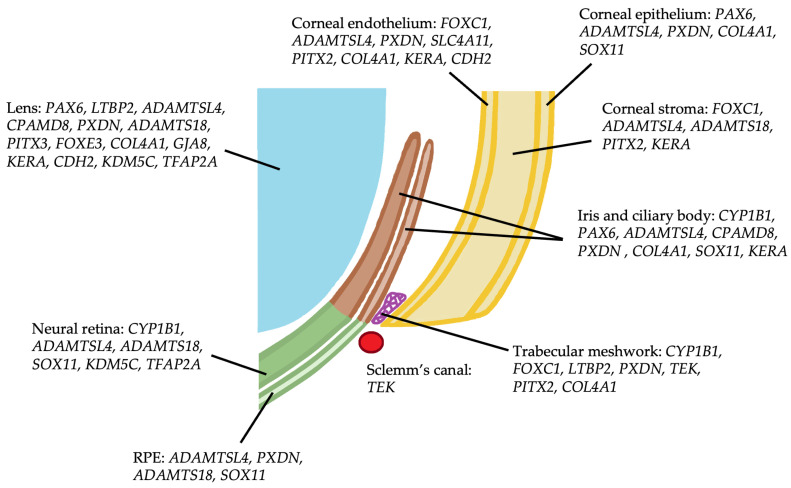
Ocular expression of genes causing non-syndromic childhood glaucoma and anterior segment dysgenesis.

Twenty genes linked to childhood glaucoma associated with anterior segment dysgenesis were identified, of which twelve were also reported to cause isolated childhood glaucoma. The reported phenotypes of anterior segment dysgenesis covered the full spectrum of cornea, iris, lens and mixed anomalies (Table 1). *PAX6*, *FOXC1* and *PITX2* are expressed in multiple anterior segment tissues during ocular development; other genes, such as *CYP1B1*, have more limited expression (Figure 3).

### 2.1. CYP1B1 (OMIM #601771)

*CYP1B1* encodes a 543-amino acid enzyme which is part of the cytochrome P450 superfamily and is involved in retinol [61,62], melatonin [61] and 17ß-oestradiol metabolism [62,63]. It regulates myocilin production by limiting estrogen-responsive activation of the *MYOC* promoter [63], and contributes to trabecular meshwork development through its modulation of periostin [64]. Pathogenic variants include missense, nonsense and frameshift mutations, and all have been identified throughout the gene [65]; the majority result in loss of function of metabolic activity [62,66,67], although some variants, such as c.1154C>A p.(Phe261Leu) and c.1776C>T p.(Arg469Trp) cause significantly increased retinol metabolism [62]. *CYP1B1* is the most commonly identified genetic cause of primary congenital glaucoma (PCG) [65,68], causing 15–35% of PCG in European studies [12,66], and up to 100% of PCG in some ethnic groups [69]. To date, there have been 62 *CYP1B1* variants associated with anterior segment dysgeneses, including the following: c.1064_1076del p.(Arg355Hisfs*69), c.1103G>A p.(Arg368His) and c.1169G>A p.(Arg390His) associated with Axenfeld–Rieger syndrome and Peters anomaly [25,28,29,31]; c.171G>A p.(Trp57*) and c.868dupC p.(Arg290Profs*37) associated with Peters anomaly and other corneal dystrophies [25,31,33,70]; and c.434_443del p.(Arg145Profs*4), c.756C>A p.(Asn252Lys) and c.1453T>C p.(Ser485Phe) associated with aniridia [30,71].

### 2.2. FOXC1 (OMIM #601090)

*FOXC1*, part of the forkhead box family of transcription factors, is expressed in the periocular mesenchyme and is necessary for separation of the cornea and lens in the developing eye [2,5,72]. It acts in conjunction with *PITX2* to promote the development of the corneal stroma and endothelium, iridocorneal angle and choroid, with co-expression of both transcription factors necessary for normal differentiation of these cellular populations [73]. In the trabecular meshwork, *FOXC1* activity is mediated by miR-204 and regulates *MEIS2* [74], a homeobox transcription factor which in turn is a direct regulator of *PAX6* [75]. *FOXC1* variants have been identified in patients with childhood glaucoma associated with Axenfeld–Rieger anomaly [15,28,34], Peters anomaly [13,33] and aniridia [13]. In the context of Axenfeld–Rieger syndrome, *FOXC1* variants cause significantly more congenital glaucoma than do *PITX2* variants, which are more associated with juvenile- and adult-onset glaucoma [15]. Whole gene deletions account for approximately one third of anterior segment dysgenesis caused by *FOXC1* [12,15]. There are 126 intragenic *FOXC1* variants associated with Axenfeld–Rieger syndrome, of which c.482T>A p. (Met161Lys) was most commonly identified [15]. In a functional study, Medina-Trillo and colleagues showed that *FOXC1* variants can result in either significantly increased or decreased transcriptional activity compared with the wild type; hypermorphic variants including c.141C>G p.(Tyr47*) and c.316C>T p.(Gln106*) were associated with isolated dominant glaucoma, whilst hypomorphic variants including c.377T>G p.(Ile126Ser) were identified in patients with Axenfeld–Rieger anomaly [76].

### 2.3. PAX6 (OMIM #607108)

Heterozygous pathogenic variants in *PAX6* are typically associated with autosomal dominant aniridia, or are part of the Wilms tumor-aniridia-genital anomalies-range of developmental delay (WAGR) syndrome if the whole gene is deleted along with *WT1* [11,18]. The PAX6 transcription factor is known to have a role in ocular development from gestational day 22 in the differentiation of the eye field and optic vesicle, through to the development of the optic cup and lens vesicle at day 37, and then in corneal, iris, ciliary body and trabecular meshwork development from day 47 onwards [2,77,78]. It has both paired and homeobox DNA-binding domains, and interacts with numerous other genes during this process, including *SOX2*, *RAX*, *MEIS2* and *ALDH1A3* [5,75,77]. Close regulation of *PAX6* expression is required throughout embryogenesis to ensure normal development; underexpression results in the aniridia spectrum of iris, foveal and optic nerve hypoplasia with associated glaucoma, cataracts and keratopathy, whilst overexpression causes microphthalmia, microcornea and ciliary body maldevelopment [79,80,81]. Of the 467 pathogenic and likely pathogenic *PAX6* variants reported to date [82], c.649C>T p.(Arg203*), c.760C>T p.(Arg240*), c.823C>T p.(Arg261*) and c.991C>T p.(Arg317*) are the most commonly identified [14]. Whilst there are reports implicating *PAX6* in isolated juvenile open angle glaucoma and childhood glaucoma associated with Peters anomaly, childhood glaucoma caused by *PAX6* variants is very rarely diagnosed in patients without aniridia [33,83].

### 2.4. LTBP2 (OMIM #602091) and ADAMTSL4 (OMIM #610113)

Both *LTBP2* and *ADAMTSL4* contribute to cytoskeletal development through their interactions with fibrillin-1. *LTBP2* encodes a 1821-amino acid extracellular matrix protein which interacts with fibrillin-1-containing microfibrils [84,85] and is expressed in the cornea, trabecular meshwork and lens [85]. A causative gene for Weill–Marchesani syndrome [28,36,84], which presents with short stature, brachydactyly, microspherophakia, ectopia lentis and myopia, *LTBP2* has also been linked with congenital glaucoma associated with isolated microspherophakia [16], ectopia lentis (c.4825T>A p.(Cys1609Ser) and c.529T>C p.(Trp177Arg) variants) [37] and congenital cataracts (c.895C>T p.(Arg299*) variant) [12]. The glycoprotein ADAMTSL4 binds fibrillin-1 and facilitates microfibril assembly, and is expressed in many ocular tissues including the cornea, iris and ciliary body in the anterior segment, and the retinal ganglion cells, retinal pigment epithelium, choroid and sclera in the posterior segment [86]. The missense variant c.2236C>T p.(Arg746Cys) has been linked to congenital glaucoma with ectopia lentis [38].

### 2.5. CPAMD8 (OMIM #608841)

*CPAMD8* is expressed in the human eye from gestational week 9 onwards [87]. From weeks 9 through 22 of gestation, *CPAMD8* expression increases in the lens and decreases in the retina, and strong expression is also observed in the iris and cornea at week 22 [87]. The pathways by which *CPAMD8* acts to contribute to ocular development remain undefined, but recent work by Escribano and colleagues in zebrafish suggests an interaction between *cpamd8* and *adamtsl4* [88], implicating a potential contribution of *CPAMD8* to the development of fibrillar structures in the anterior segment. *CPAMD8* variants have been linked to anterior segment dysgenesis associated with both congenital and juvenile glaucoma [16,39,40,41], with the variants c.4298C>A p.(Thr1433Asn) and c.2352dupC p.(Arg785Glnfs*23) identified in more than one family [40].

### 2.6. COL4A1 (OMIM #120130) and PXDN (OMIM #605158)

Variants in *COL4A1* and *PXDN*, which are both basement membrane components, have been identified in patients with anterior segment dysgeneses associated with childhood glaucoma, although only the c.2159G>A p.(Gly720Asp) has been reported in more than one family [13,33,42,43,55,56]. *COL4A1* encodes the alpha-1 subunit of type IV collagen, which is the main component of endothelial and epithelial basement membranes [89]. Peroxidasin, the protein product of the *PXDN* gene, forms sulfilimine crosslinks between type IV collagen molecules, and is necessary for normal development of type IV collagen-, laminin- and fibronectin-based fibrillar networks in endothelial cells [90]. Type IV collagens are found throughout the developing eye, and by embryonic day 18.5 (E18.5), the alpha-1 isoform can be identified in all anterior segment tissues in mice [91].

### 2.7. TEK (OMIM #600221)

One of two receptor tyrosine kinases in the angiopoietin/TEK signaling pathway, autosomal dominant mutations in *TEK* have been identified in patients with primary congenital glaucoma [16] and congenital glaucoma associated with sclerocornea (specific variant not reported) [36]. In mice, *TEK* is expressed in Schlemm’s canal endothelial cells, and its ligand *Angpt1* is expressed in the trabecular meshwork [92,93]. Supporting the role of *TEK* in iridocorneal angle and Schlemm’s canal development, murine studies have shown that *Tek* haploinsufficiency and *Angpt1* knockout result in severe hypoplasia of both Schlemm’s canal and the trabecular meshwork, whilst *Tek* knockout results in complete failure of Schlemm’s canal development [93,94].

### 2.8. SLC4A11 (OMIM #610206)

Pathogenic variants in *SLC4A11* are the most commonly identified genetic cause of congenital hereditary endothelial dystrophy (CHED) [95], which presents with early onset corneal edema and, rarely, concomitant congenital glaucoma [16,32,46]. Homozygous missense variants cause approximately half of *SLC4A11*-related disease [95], and both families with congenital glaucoma whose variants have been reported had homozygous missense mutations; these were c.1343G>A p.(Gly448Asp) and c.2024A>C p.(Glu657Ala), respectively [32,46]. *SLC4A11* localizes to the corneal endothelium, where it contributes to osmotic homeostasis of the cornea through its action as a transmembrane sodium-coupled hydroxyl transporter, and thus to maintenance of corneal transparency [95,96]. *Slc4a11*-knockout mice develop progressive corneal thickening with associated endothelial cell morphological changes and loss [97], but no groups have yet reported intraocular pressure in these murine models.

### 2.9. ADAMTS18 (OMIM #607512)

Part of the ADAMTS zinc-dependent metalloproteinase family, *ADAMTS18* is expressed in adnexal, anterior segment and retinal cells during murine ocular development, with expression persisting postnatally in the lens and lacrimal gland [98,99]. It is a key factor in branching morphogenesis of the embryonic lacrimal gland [98], as well as in the development of the lung, breast and reproductive tract [24]. In relation to anterior segment development, it interacts with fibrillin-1 and is modulated by estrogen, raising the possibility of an interaction with *CYP1B1* [24]. Pathogenic variants in *ADAMTS18* cause microcornea, myopic chorioretinal atrophy, and telecanthus (MMCAT) [100] and Knobloch syndrome (high myopia, vitreoretinal degeneration, retinal detachment, cataract and occipital encephalocele) [101], but childhood glaucoma associated with microcornea has been reported in twins with compound heterozygous missense mutations of *ADAMTS18* [c.3436C>T p.(Arg1146Trp) and c.1454T>G p.(Phe485Cys)] identified through panel testing [47]. Ocular defects seen in *Adamts18* knockout mice include breaks in the lens capsule resulting in extrusion of material, but microphthalmia, microcornea and retinal anomalies were not seen [99]. However, morpholino oligonucleotide (MO) knockdown of *adamts18* in zebrafish has been shown to cause macroscopic ocular defects [102].

### 2.10. PITX2 (OMIM #601542) and PITX3 (OMIM #602669)

The bicoid-type homeodomain transcription factors *PITX2* and *PITX3* have a role in embryonic anterior segment and lens development [103,104]. As discussed above, *PITX2* interacts with *FOXC1*, and in mice, they are co-expressed in the periocular mesenchyme from E11.5 [73]. It is therefore unsurprising that the ocular phenotypes of *PITX2*-related disease are similar to those of *FOXC1* and include childhood glaucoma associated with Axenfeld–Rieger anomaly [16,33] and Peters anomaly [13,16]. As with *FOXC1*, the majority of *PITX2* variants have been identified in only one or two families, with the exception of the splicing variant c.253-11A>G and the intragenic variants c.191C>T p.(Pro64Leu), c.356del p.(Gln119Argfs*36) and c.475_476del p.(Leu159Valfs*39), which have all been reported in four or more families [15]. *PITX3* is expressed in the lens placode at murine E10 and continues to be expressed in the lens throughout development, in addition to expression in the midbrain, tongue and other embryological craniofacial tissues [104]. The c.640_656dup p.(Gly220Profs*95) variant has been identified in two families with congenital glaucoma associated with microcornea and congenital cataracts [48,49].

### 2.11. FOXE3 (OMIM #601094)

*FOXE3* variants are associated with a wide spectrum of anterior segment phenotypes and have been identified in patients with childhood glaucoma associated with Peters anomaly [50,51,52], congenital cataracts/aphakia [51,53] and microphthalmia [51,54]. The most common variants are c.21_24del p.(Met7Ilefs*216), c.244A>G p.(Met82Val) and c.720C>A p.(Cys240*) which have all been reported in four or more families [51]. The main role of the FOXE3 transcription factor during embryogenesis is in the development of the lens, where it is expressed in mice from E12.5 onwards and directly regulates the autophagy-associated protein DNAJB1 [50]. Functional studies of *FOXE3* missense variants and small deletions isolated from patients with recessive disease showed significantly reduced transcriptional activity with homozygous or compound heterozygous variants, but no disease phenotype in their heterozygous parents, indicating that the presence of a single functional allele is sufficient for normal anterior segment development [53].

### 2.12. SOX11 (OMIM #600898)

The SOX11 SRY-Box transcription factor is a close homolog of SOX2 [105], which is known to play a key role in ocular development from induction of the eye field at gestational day 21 onwards [77]. During *Xenopus* embryogenesis, *sox11* is expressed in the optic vesicle, retina and anterior segment, and MO knockdown of *sox11* results in decreased *pax6* expression in the eye field, loss of retinal lamination and a microphthalmic phenotype [106]. In *Sox11* knockout mice, *Pax6* expression is unaffected, but *BMP7* expression is reduced, and the phenotype includes lenticular defects, Peters anomaly and anterior ocular coloboma [107]. *SOX11* variants, including c.251G>T p.(Gly84Val), have been reported in patients with congenital glaucoma associated with Peters anomaly and aniridia [36,57], although the gene is more commonly associated with intellectual developmental disorder with microcephaly with or without ocular malformations or hypogonadotropic hypogonadism (IDDMOH; Coffin Siris syndrome), of which congenital glaucoma can be a feature [28,57].

### 2.13. GJA8 (OMIM #600897)

*GJA8* encodes connexin 50 (Cx50), a gap junction protein of lens fiber cells which is necessary for maintaining lens transparency and cellular homeostasis [28,108]. Studies of the missense mutation c.217T>C p.(Ser73Pro) in transfected human lens epithelial cells have shown aggregation of the abnormal protein in the cytosol with loss of localization to the plasma membrane, resulting in reduced function of gap junction channels and increased apoptosis [108]. Although *GJA8* variants are more commonly associated with congenital cataracts [11], the missense variants c.280G>C p.(Gly94Arg) and c.151G>A p.(Asp51Asn) have been identified in patients with congenital glaucoma associated with sclerocornea [28,33,36].

### 2.14. KERA (OMIM #603288)

*KERA* encodes keratocan, a keratan sulfate proteoglycan of the corneal stroma with a central role in maintaining collagen spacing and thus corneal transparency through its interaction with keratan sulfate glycosaminoglycans and collagen fibrils [109,110]. In adults, it is localized to stromal keratocytes [109]; during embryological development, studies of chicks by Conrad [111] and Gealy [112] have shown expression of *Kera* in the periocular mesenchyme from E4.5, with subsequent localization to the anterior corneal stroma. Keratocan-deficient mice have a thinner cornea and narrower iridocorneal angle than wild-type mice, with larger collagen fibrils in a less regular arrangement [110]. Mutations in *KERA* are predominantly linked to cornea plana, in which there is flattening of the normal convex curvature of the cornea resulting in hyperopia [113], but one patient with anterior segment dysgenesis and congenital glaucoma associated with a homozygous nonsense *KERA* mutation c.937C>T p.(Arg313*) has been reported [39].

### 2.15. CDH2 (OMIM #114020), KDM5C (OMIM #314690) and TFAP2A (OMIM #107580)

*CDH2, KDM5C* and *TFAP2A* are involved in neural development, with roles including axonal pathfinding [114], neuronal differentiation [115] and neural crest migration [116], respectively. All are reported to be causative of congenital glaucoma associated with Peters anomaly [58,59,60]. *CDH2* encodes N-cadherin, part of the cadherin family of Ca^2+^-dependent transmembrane glycoproteins which regulate cell–cell adhesion in multiple tissues [58,114]. *CDH2* is expressed in murine lens cells and the lens stalk at E10.75-11.0, and in lens and corneal endothelial cells at E17.5 [58]. In conjunction with *CDH1* and *CDH3,* it controls separation of the lens vesicle from the surface ectoderm during anterior segment development; thus, its association with Peters anomaly is unsurprising [117]. Knockout of N-cadherin from the lens vesicle in mice results in a spectrum of anterior segment anomalies, including severe iris hyperplasia, microphthalmia and small, underdeveloped lenses with abnormal lens fiber development [117]. The missense variants c.485T>A p.(Val162Asp), c.1574A>G p.(Asp525Gly) and c.1807C>T p.(Pro603Ser), and splicing variant c.702+1G>A, have each been identified in one patient with Peters anomaly and congenital glaucoma [58].

*KDM5C* is a cause of X-linked syndromic intellectual developmental disorder, but the missense variant c.1204G>A p.(Asp402Asn) has been identified in one patient with Peters anomaly and congenital glaucoma [59]. Involved in transcriptional regulation through demethylation of H3K4 residues, studies of *kdm5c* in *Xenopus* have revealed that it is strongly expressed throughout neural and ocular development, particularly in the lens and retina [118]. MO knockdown studies in *Xenopus* have confirmed the essential role of *kdm5c* in ocular development, with loss of *kdm5c* resulting in microphthalmia, coloboma and abnormal retinal lamination [118].

In human eyes, expression of the transcription factor *TFAP2A* is seen in the anterior lens epithelium from gestational day 35 and in the equatorial lens epithelium, secondary lens fibers and neural retinal by day 54 [119]. Whilst the detailed mechanism by which *TFAP2A* acts has not yet been elucidated, conditional knockout of *Tcfap2a* in the lens placode of mice has been shown to result in failure of lens separation from the surface ectoderm with associated corneal anomalies, accompanied by loss of *Foxe3* and *Pitx3* expression from the lens stalk, suggesting that *TFAP2A* may be an upstream regulator of these genes as they pertain to lens separation [120]. This is consistent with the identification of a *TFAP2A* splicing variant (c.1025+2T>A) in a patient with Peters anomaly associated with congenital glaucoma [60].

## 3. Management of Childhood Glaucoma and Anterior Segment Dysgenesis

The current treatment options for anterior segment dysgenesis and childhood glaucoma are predominantly surgical, although refractive, medical and supportive interventions, including genetic counseling and family planning, all contribute to the holistic management of these patients and their families [17,79,121]. For congenital glaucoma, angle surgery (goniotomy and trabeculotomy) is the first line treatment; trabeculectomy with anti-fibrotic agents, glaucoma drainage devices and cyclodestructive procedures such as transscleral cyclophotocoagulation are used in refractory cases. Medical treatments (beta-blockers, carbonic anhydrase inhibitors and prostaglandin analogs) are used as adjuncts to surgery or as temporizing measures while awaiting a procedure [121,122]. In patients with aniridia, cataract surgery, and corneal and limbal grafts, alongside ocular lubricants, refraction and treatment of associated glaucoma have long been the mainstay of treatment [30,79]. Many patients with Peters anomaly require penetrating keratoplasty, although newer grafting techniques such as Descemet stripping automated endothelial keratoplasty (DSAEK) have been successfully employed in selected patients, and optical iridectomy may improve vision in patients with a smaller area of corneal opacity [17,123]. In all cases, refraction and amblyopia management should be provided as necessary, alongside educational and family support. Examination under anesthetic should be considered for young children to enable detailed examination and identification of subtle signs of anterior segment dysgenesis or associated ocular conditions. Genetic testing is paramount to enable accurate genetic counseling, family planning advice and referral to other specialties in cases that may have an associated syndromic element. Furthermore, as the potential for molecular-based therapies is beginning to translate into practice with promising research including developments in gene editing and nonsense suppression drugs, genetic testing will allow patients access to targeted research.

## 4. Molecular Therapies Under Development

There are currently 41 FDA-approved cellular and gene therapies [124], of which only Luxturna (voretigene neparvovec-rzyl) targets an ocular condition, *RPE65*-related retinal dystrophy. Growing research into this field as a potential source of therapy for glaucoma and anterior segment disorders has focused on animal and in vitro models, but in recent years, this has begun translating into clinical trials [79,125]. Most of these studies have investigated treatments for corneal diseases and the various methods of gene delivery. Transduction of target cells with recombinant adenoviruses (rAds) and adeno-associated viruses (rAAVs) are the most common viral vectors [125,126,127], although retroviruses [128] and lentiviruses [129] have been used in some studies. Non-viral vectors include metal and magnetic nanoparticles, micelles and lipid nanoparticles [125,130,131,132], which may be delivered topically or via injection. Alternative methods that do not require a vector include corneal electroporation and iontophoresis [133,134]. In the anterior segment, research into gene-based therapies has focused predominantly on corneal and ocular surface disease, adult-onset glaucoma and aniridia [79,125,135,136], with no published trials of gene therapies for Peters anomaly or Axenfeld–Rieger syndrome. Here, we summarize the current research into cellular and gene-based therapies for glaucoma and aniridia, and discuss the challenges faced by these approaches in the pediatric population.

### 4.1. Gene-Based Therapies for Glaucoma

Molecular-based therapies under development for glaucoma are predominantly targeted towards modification of the aqueous production/outflow pathways and minimizing damage to retinal ganglion cells (RGCs) [135], as these are key factors in the development of multifactorial adult-onset glaucoma. Sulak and colleagues [136] identified 153 studies into gene-based therapies for glaucoma and noted that there were twice as many therapies targeting RGC neuroprotection compared with those targeting aqueous production and outflow. Strategies to modulate the molecular pathways involved in aqueous regulation are in development, with targets including *MYOC*, *AQP1* and matrix metalloproteinases (MMPs).

Mutations in the *MYOC* gene, encoding the extracellular matrix protein myocilin, are the most commonly identified single gene cause of primary open angle glaucoma (POAG) and juvenile open angle glaucoma (JOAG), identified in 2–4% and 10–30% of cases, respectively [135]. Dominant gain-of-function mutations result in intracellular accumulation of the abnormal protein, resulting in increasing intraocular pressure (IOP) and resultant glaucomatous damage to the retinal ganglion cells and optic nerve [127]. Mice with the *MYOC* p.(Tyr437His) variant (Tg-*MYOC^Y437H^*) can be used as a model for POAG, developing these same pathogenic features, and may be employed for in vivo confirmation of the therapeutic benefit [127,129].

Gene editing approaches targeted at specific myocilin mutations are in development. Jain [127] used a clustered regularly interspaced palindromic repeats (CRISPR)-CRISPR-associated protein 9 (Cas9) approach with an adenoviral Ad5 vector to introduce a truncating frameshift mutation into Tg-*MYOC^Y437H^* mice. They demonstrated significant reduction in IOP into the normal range in both younger (age ≤ 1 month) and older (age ≥ 9 months) mice, with results sustained for at least 2 months post-injection. The group have subsequently shown positive results using the same CRISPR-Cas9 system with a lentiviral vector [129], with IOP reduction into the normal range in Tg-*MYOC^Y437H^* mice maintained for at least 6 weeks post-injection. They additionally tested an AAV2 vector in vitro on human TM3 cells with p.(Tyr437His) or p.(Gly364Val) *MYOC* mutations, but the reduction in accumulated myocilin was only 34% lower than untreated levels with the AAV2 vector compared with 62% reduction for the lentivirus. A non-randomized interventional trial of a BD113 virus-like particle, a lentiviral-based CRISPR-Cas9 gene therapy targeted to knock down *MYOC*, has commenced recruitment of adults with myocilin-related POAG refractory to initial treatment and will primarily assess IOP reduction following a single intracameral injection [137]. Another mechanism that has been proposed for reducing the pathogenicity of *MYOC* mutations is to use small interfering RNAs (siRNAs) to disrupt the expression of the gene. The preliminary results in TM5 cells demonstrated significant suppression of both wild-type and mutant myocilin expression to 60% of control levels [138]. Whilst further work is needed, these approaches hold promise for potential gene-targeted therapies for *MYOC*-related glaucoma to become available in the future.

Another CRISPR-based approach employing a lentivirus vector carrying single guide RNA (sgRNA) to target the *TGFβ2* promoter has been developed by Mao’s group [139]. Administration of the sgRNA lentivirus 2.5 weeks prior to induction of ocular hypertension prevented IOP increases, maintaining an IOP level below 20 mmHg for at least 8 weeks post-injection, compared with an increase to over 30 mmHg in controls. *AQP1*, which encodes aquaporin 1, has also been targeted with sgRNA. This transmembrane water channel is present in many tissues including the ciliary body, and deletion of *Aqp1* has been shown to significantly reduce IOP in mice [140]. Introduction of two *AQP1*-indel-inducing sgRNAs within a single AAV vector given by intravitreal injection has been shown to transduce the murine ciliary body and reduce IOP by 22% after 3 weeks, with neuroprotective effects sustained to at least 7 weeks post-injection [141]. There was also effective transfection of ex vivo human ciliary body epithelial cells. *AQP1* has therefore been proposed as a potential therapeutic target for glaucoma.

MMP activity is known to be reduced in glaucoma, and one of the key actions of the prostaglandin analog drops used to treat open angle glaucoma is to increase MMP expression in the ciliary body [142]. Using an AAV9 vector to transduce corneal endothelial cells, O’Callaghan [143] introduced tetracycline-inducible murine MMP3 into Tg-*MYOC^Y437H^* and dexamethasone-induced ocular hypertensive mice. In both groups, IOP 2 weeks after doxycycline induction was significantly reduced in the treated mice compared with controls. They also demonstrated significant increases in MMP3 expression and aqueous outflow in non-human primates in vivo, and increased aqueous outflow in human donor anterior segments in vitro using this approach [143]. Similar effects have been achieved using intracameral IκBα-silencing siRNA to transfect the anterior segment of nonhuman primates, with upregulation of MMP2 and MMP9 and resultant 49% reduction in IOP [144]. A lesser IOP reduction has been achieved through the use of the microRNA miR-21-5p, which is predicted to have downstream effects including the activation of MMP9. Whilst preliminary results show potential, the maximal IOP reduction following administration was 17.8% [145], lower than the 20–35% achieved by applying current topical treatments [146].

Novel gene-targeted approaches acting on established therapeutic pathways are also under development. Rho kinase inhibitors act directly on the trabecular meshwork to inhibit phosphorylation of proteins including myosin light chains, which reduces trabecular cell adhesion and resistance and promotes phagocytosis of extracellular debris by trabecular reticulum cells, thus increasing aqueous outflow and reducing IOP [147]. Current commercial formulations are administered topically, but a Phase 1 study of GVB-2001 gene therapy in adults with POAG is open to recruitment [148]. GVB-2001 consists of a human RhoA gene with a dominant negative mutation carried in an AAV vector, an approach that has been successful in transducing the trabecular meshwork and reducing nocturnal IOP rises in rats through inhibition of the Rho pathway [149].

Targeted disruption of β2-adrenoceptors has also shown promise. Loma [146] topically administered siRNAs designed to silence β2-adrenoceptors to rabbits and compared the effects to commercially available topical dorzolamide, latanoprost and timolol. The siRNAs significantly reduced IOP by 30 ± 5% compared to controls, with effects detectable 24 h after instillation and sustained for 5 days. The level of IOP reduction was comparable to that achieved by all commercial agents, but with a longer onset and offset time. A commercial compound, bamosiran, has recently completed its phase II trial. This RNA interference approach designed to prevent β2-adrenoceptor production showed non-inferiority to timolol in patients with a baseline IOP ≥ 25 mmHg, but not across all patient groups [150]. However, the positive results achieved overall by using these diverse approaches to molecular modulation of aqueous regulation lay a strong foundation for molecular therapies to become available for patients within the near future.

### 4.2. Gene-Based Therapies for Aniridia

A novel approach to the treatment of aniridia is nonsense suppression therapy, using drugs that impair recognition of premature termination codons (PTCs) during translation to allow the production of a full-length protein [151]. In aniridia patients in whom a genetic cause is identified, dominant pathogenic *PAX6* mutations are responsible for approximately 90% of cases [14]; of these, nonsense and frameshift mutations resulting in a PTC comprise 70% of pathogenic and likely pathogenic *PAX6* variants [82], so a treatment that targets these mutations has the potential to benefit a large proportion of these patients. Promising results using nonsense suppression therapies, which work on in-frame nonsense mutations, have been reported in vitro [152] and in the *Sey* mouse model [153,154]. *Sey* mice with a heterozygous *PAX6* p.(Gly194*) nonsense mutation treated with topical nonsense suppression drugs from postnatal day 14–60 developed partial phenotypic rescue, including reversal of corneal, lens and retinal abnormalities [153,154]. However, these benefits have not yet been translated into positive results in clinical trials. At completion, the Study of Ataluren in Participants With Nonsense Mutation Aniridia (STAR) unfortunately failed to meet its primary endpoint, with no significant difference in change in reading speed at 48 weeks between the treatment and placebo groups [155]. STAR used an oral formulation of ataluren; a topical alternative has been developed with proven physical stability and microbiological sterility over 60 days [156] which may have better ocular bioavailability and improved efficacy, and the outcomes from these assessments are awaited.

Gene editing is under investigation as another potential avenue of treatment for aniridia. Work in *Sey* mice has shown successful germline correction of the *PAX6* p.(Gly194*) mutation in vivo via microinjection of the targeted CRISPR-Cas9 complex into murine zygotes at day 0.5 post-coitus, with inheritance of the corrected gene by the offspring of these transgenic mice [157]. The progeny had normal eye development and normal *PAX6* levels in neural tissue at embryonic day 18.5, when microphthalmic changes are observed in *Sey* mice [157]. Further work has demonstrated the potential use of AAV vectors for delivery of gene therapy in aniridia, with results suggesting that the AAV9 capsid is able to safely transduce corneal and retinal cells of *Sey* mice [126].

Work to expand the therapeutic options for management of aniridia-associated keratopathy has shown that duloxetine and ritanserin, both anti-serotonin drugs which inhibit the MEK/ERK (mitogen-activated protein kinase-ERK kinase/extracellular signal related kinase) signaling pathway, can enhance endogenous *PAX6* expression in haploinsufficient corneal limbal epithelial stem cells [158,159,160]. A regulatory microRNA, miR-204-5p, has also been reported that upregulates *PAX6* in *PAX6*-knockdown corneal limbal stem cells and mature differentiated corneal epithelial cells in vitro; there was no significant change in *PAX6* expression in primary human limbal epithelial cells, human limbal stem cells or in the corneas of *Pax6*^(*Sey/+*)^ mice transfected with miR-204-5p [161]. Further work is needed to determine the effects of these modulating factors in other ocular cell types, but they provide another potential avenue for therapeutic development. However, any strategies to increase *PAX6* expression during embryogenesis need to ensure that induced levels remain carefully modulated. Both under- and overexpression of *PAX6* can lead to ocular developmental anomalies as discussed earlier [80,81]; therefore, ensuring that corrected *PAX6* expression levels are as close as possible to the normal embryological state is essential when correcting pathogenic variants. To address this, Simpson’s team [162] have designed *PAX6* MiniPromoters and reported their robust and specific expression within the *PAX6*-positive RGC, amacrine, horizontal and Müller glial cells, with significantly better regulation of *PAX6* expression than achieved by the ubiquitous smCBA promoter.

### 4.3. Challenges of Gene Therapy in the Pediatric Population

The anterior segment dysgeneses are congenital conditions with phenotypic features resulting from abnormal oculogenesis. Consideration therefore needs to be given to the challenges presented when administering any molecular therapies to a pediatric population and determination of trial endpoints suited to this group.

The first challenge is confirming the genetic diagnosis. Whilst molecular diagnostic rates for aniridia now reach 90–98% [163,164], rates for other ASDs remain much lower at less than 25% [12,23]. For gene-based therapies, accurate molecular diagnosis is essential, particularly given the overlapping phenotypes caused by many of the genes listed above. Secondly, these are rare diseases; therefore, identification of a sufficient number of patients for clinical trials may require collaboration across multiple centers and potentially multiple countries. Local and national rare disease registries and collaboration with charitable networks can aid in this.

Trial endpoints need to be carefully considered. Appropriate functional tests of acuity and fields vary with age, and it may be difficult to acquire quantitative results [165]. For IOP testing, Goldmann applanation tonometry remains the gold standard [166] but requires the patient to sit at the slit lamp. IOP testing with handheld devices such as the iCare tonometer can allow for monitoring in children of all ages, but their accuracy at pressures above 23 mmHg is reduced [166]; therefore, their use in trials of patients with childhood glaucoma needs to be carefully considered. For ASDs, monitoring of phenotypic changes may be necessary, and in some children, this may require examination under anesthesia, which should be incorporated into routine clinical care wherever possible.

Finally, there are the challenges inherent in the gene therapies themselves. Intracameral administration of therapies targeting the corneal endothelium and trabecular meshwork negates the need for corneal penetrance required by topical formulations and reduces the potential for systemic off-target effects, but this method of delivery to pediatric patients requires general anesthesia and introduces the potential for intraocular infection. Adenoviral vectors can provoke immune responses and inflammation whereas AAVs and lentiviruses rarely elicit these reactions [125]. Although less likely than with retroviruses, concerns have been raised regarding the potential for lentiviral vectors to cause insertional mutagenesis, or for additional mutagenic effects to occur with CRISPR-Cas9 delivery systems [125], but Jain [127] identified no alternative myocilin isoforms following treatment with a CRISPR-Cas9 approach. Studies of transduction of target cells in vitro have been promising, with up to 70% transduction efficacy of human trabecular meshwork cells by an Ad5 vector using CRISPR-Cas9 to target mutant *MYOC,* and reduction in IOP using this therapy in Tg-*MYOC^Y437H^* mice [127]. However, exposure to AAVs in childhood is common and therefore pre-existing circulating antibodies may reduce transduction efficacy in human subjects [165].

### 4.4. Future Research Directions

Future research directions in this field should focus on both targeting the underlying biological pathways affected and on improving the delivery methods in the pediatric population. To date, aniridia and adult-onset glaucoma have been the primary foci for therapeutic development, but the approaches developed in these fields have the potential to be applicable to other anterior segment dysgeneses with a similar genetic basis. For example, 13% of known pathogenic variants of *CYP1B1* are nonsense mutations, but these account for 21% of *CYP1B1*-related anterior segment dysgenesis. Studies of nonsense suppression therapies in aniridia have shown promising results, with partial phenotypic rescue in mice treated post-partum; similar trials in animal models of other nonsense-mediated anterior segment dysgeneses are an avenue that warrants further exploration. Likewise, whilst primary congenital glaucoma is thought to be predominantly driven by iridotrabeculodysgenesis and changes in Schlemm canal development, the juvenile-onset glaucoma seen in aniridia and Axenfeld–Rieger syndrome may be amenable to modulation of *TGFβ2*, *AQP1* or MMPs. If these treatments progress smoothly through Phase 1 and 2 trials in adult-onset glaucoma, trials in glaucoma secondary to anterior segment dysgeneses should be considered.

## 5. Methods

A combination of strategies was used to identify the genes that cause non-syndromic childhood glaucoma associated with anterior segment dysgenesis. A list was compiled in June 2024 of all green and amber genes in the NHS Genomics Medicine Service’s “Structural Eye Disease” panel (v4.1) and all genes and conditions associated with the term “glaucoma” in Online Mendelian Inheritance in Man (OMIM) [167]. Each gene identified was then searched on PubMed for reports and studies detailing its association with childhood glaucoma and any anterior segment developmental anomaly. Any additional genes identified through these publications which were reported to be associated with childhood glaucoma and any anterior segment developmental anomaly were also reviewed. Inclusion criteria: all genes with a confirmed causative link to glaucoma onset under 18 years of age associated with any anterior segment developmental anomaly without syndromic features. Exclusion criteria: any genes reported only in patients with isolated childhood glaucoma, syndromic ASD or in whom glaucoma was identified subsequently to cataract surgery.

## 6. Conclusions

As the accessibility of genetic testing for ocular diseases has increased, so too has the number of genes identified that are linked to childhood glaucoma associated with anterior segment dysgenesis, although the molecular diagnostic rates for these conditions remain low. To date, twenty genes have been associated with this subset of ocular developmental disorders, with many more involved in the pathogenesis of primary congenital glaucoma and anterior segment dysgenesis without glaucoma [11]. The pathways governing anterior segment development are complex and mutations in different genes can cause very similar clinical features. Furthermore, there is significant variation in the phenotype resulting from mutations within individual genes. Improvements in individualized patient prognostication and management will benefit from anatomical-based reporting of clinical features and gene-based diagnosis, both for identifying the most appropriate therapies and allowing detailed genotype–phenotype correlation studies of these rare conditions in the future. Additionally, it is a step towards the change in approach to treatment required in this advent of gene therapy. As the development of these targeted treatments begins to translate into clinically available management options, knowing a patient’s underlying genetic diagnosis will be key to ensuring that they receive appropriate treatment.

## Figures and Tables

**Table 1:** Genes reported to be linked to childhood glaucoma associated with anterior segment dysgenesis, and the phenotypes identified in each. AD = autosomal dominant, AR = autosomal recessive.

Gene	OMIM ID	Phenotype(s) of Anterior Segment Dysgenesis Associated with Childhood Glaucoma	Inheritance Pattern	Associated with Isolated Childhood Glaucoma
*CYP1B1*	601771	Peters anomaly [25,26,27,28] Axenfeld–Rieger anomaly [29] Aniridia [30] Corneal dystrophy [12,31] Unclassified ASD [32].	AR	Yes
*FOXC1*	601090	Peters anomaly [13,33] Axenfeld–Rieger anomaly [15,28,34] Aniridia [13] Unclassified ASD [12,13].	AD	Yes
*PAX6*	607108	Aniridia [16,34,35] Peters anomaly [33].	AD	Yes
*LTBP2*	602091	Weill–Marchesani syndrome [28,36] Congenital cataracts [12] Lenticular anomalies [16,37] Unclassified ASD [16].	AR	Yes
*ADAMTSL4*	610113	Ectopia lentis [38].	AR	Yes
*CPAMD8*	608841	Lenticular anomalies [39,40] Iris anomalies [40] Unclassified ASD [16,41].	AR	Yes
*PXDN*	605158	Peters anomaly [42] Congenital cataracts [43] Aphakia [42] Aniridia [44] Sclerocornea [33,45].	AR	Yes
*TEK*	600221	Sclerocornea [36].	AD	Yes
*SLC4A11*	610206	Congenital hereditary endothelial dystrophy [16,32,46].	AR	Yes
*ADAMTS18*	607512	Microcornea [47].	AR	Yes
*PITX3*	602669	Microcornea [48,49] Congenital cataracts [49].	AD	Yes
*PITX2*	601542	Peters anomaly [13,16] Axenfeld–Rieger anomaly [16,33] Sclerocornea [33].	AD	No
*FOXE3*	601094	Peters anomaly [50,51,52] Iris anomalies [50,51] Congenital cataracts/aphakia [51,53] Microphthalmia [51,54].	AD/AR	No
*COL4A1*	120130	Congenital cataracts [13,55,56] Axenfeld–Rieger anomaly [56] Iris anomalies [13,55].	AD	No
*SOX11*	600898	Peters anomaly [57] Aniridia [36,57].		No
*GJA8*	600897	Sclerocornea [28,33,36].	AD	No
*KERA*	603288	Unclassified ASD [39].	AR	No
*CDH2*	114020	Peters anomaly [58].	AD	No
*KDM5C*	314690	Peters anomaly [59].	AD	No
*TFAP2A*	107580	Peters anomaly [60].	AD	No

## Data Availability

No new data were created or analyzed in this study. Data sharing is not applicable to this article.

## Abbreviations

The following abbreviations are used in this manuscript:

AD	Autosomal dominant
AR	Autosomal recessive
ASD	Anterior segment dysgenesis
BMP	Bone morphogenetic protein
CHED	Congenital hereditary endothelial dystrophy
CRISPR-Cas9	Clustered regularly interspaced palindromic repeats-CRISPR-associated protein 9
DSAEK	Descemet stripping automated endothelial keratoplasty
ECM	Extracellular matrix
FDA	US Food and Drug Administration
IOP	Intraocular pressure
JOAG	Juvenile open angle glaucoma
MEK/ERK	Mitogen-activated protein kinase-ERK kinase/extracellular signal-related kinase
MMCAT	Microcornea, myopic chorioretinal atrophy and telecanthus
MMP	Matrix metalloproteinase
MO	Morpholino oligonucleotide
OMIM	Online Mendelian Inheritance in Man
PCG	Primary congenital glaucoma
POAG	Primary open angle glaucoma
PTC	Premature termination codon
rAAV	Recombinant adeno-associated virus
rAd	Recombinant adenovirus
sgRNA	Single guide RNA
siRNA	Small interfering RNA
RGC	Retinal ganglion cell
RPE	Retinal pigment epithelium
STAR	Study of Ataluren in Participants With Nonsense Mutation Aniridia
